# Pharmacokinetics of enflicoxib in dogs: Effects of prandial state and repeated administration

**DOI:** 10.1111/jvp.12995

**Published:** 2021-06-23

**Authors:** Josep Homedes, Marta Salichs, Josep Solà, Angel Menargues, Josep‐Maria Cendrós, Gregorio Encina

**Affiliations:** ^1^ Ecuphar veterinaria SLU (Animalcare Group) Barcelona Spain; ^2^ Experimental Toxicology and Ecotoxicology Unit (CERETOX) Barcelona Science Park Barcelona Spain; ^3^ Biopharmaceutics and Pharmacokinetics Unit Department of Pharmacy and Pharmaceutical Technology and Physical‐Chemistry School Faculty of Pharmacy and Food Sciences University of Barcelona Barcelona Spain; ^4^ Welab Barcelona, Barcelona Science Park (PCB) Barcelona Spain

**Keywords:** bioavailability, dog, Enflicoxib, food, pharmacokinetics

## Abstract

The pharmacokinetics of enflicoxib were evaluated in both a bioavailability study and a multi‐dose safety study in Beagle dogs. When administered at 8 mg/kg, the oral bioavailability (F) of enflicoxib was 44.1% in fasted dogs, but F increased to 63.4% under post prandial conditions. Enflicoxib is rapidly metabolised. After the first 48 h, the plasma levels of its pyrazol metabolite were much higher and persistent than those of the parent compound. Following intravenous administration, the total body plasma clearance of enflicoxib was of 140 ml/h/kg and the volume of distribution based on the terminal phase was 4 L/kg. Plasma protein binding for both compounds was approximately 99%. The blood to plasma ratio for the pyrazol metabolite showed saturable kinetics with higher blood cell affinity at lower total blood concentrations which ranged from 2.49 to 0.95 for concentrations from 1 to 15 µg/ml. Enflicoxib and its pyrazol metabolite exhibited dose‐proportional pharmacokinetics for single oral doses of 8–40 mg⁄kg and for multiple oral doses of 4–20 mg⁄kg. After 7 months of repeated weekly administrations, pre‐dose plasma concentrations (C_min,ss_) remained constant throughout the study, with no trend to any significant over‐accumulation. The mean terminal elimination half‐life (t_½_) was 20 h for enflicoxib and 17 days for the pyrazol metabolite. The pharmacokinetic profile of enflicoxib and its pyrazol metabolite in dogs supports the proposed dosing regimen in which doses are separated by 1 week.

## 1 INTRODUCTION

**FIGURE 1:**
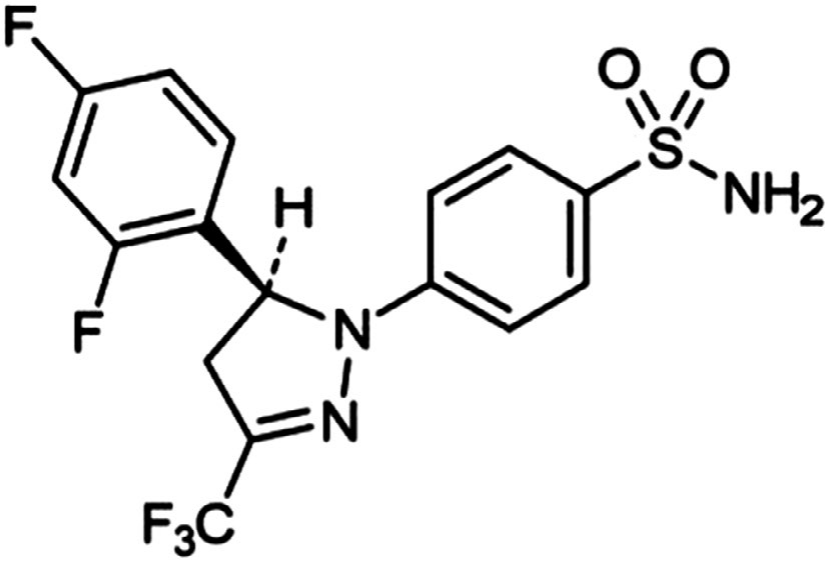
Chemical structure of enflicoxib

Enflicoxib (also known by its research acronym E‐6087) is a new non‐steroidal anti‐inflammatory drug (NSAID) of the coxib class, intended for the treatment of pain and inflammation associated with osteoarthritis in dogs (VMD, 2021) that shows potent anti‐inflammatory and analgesic activities when tested in experimental models of inflammation and pain (Wagemakers et al., 2009). Its chemical name is 4‐(5‐(2,4‐difluorophenyl)‐3‐(trifluoromethyl)‐4,5‐dihydro‐1H‐pyrazol‐1‐yl) benzenesulfonamide (C_16_H_12_F_5_N_3_O_2_S, Figure 1). After oral administration to dogs, it is readily absorbed and undergoes extensive hepatic metabolism forming two main phase I metabolites, a hydroxylated pyrazoline and a pyrazol derivative. Both enflicoxib and its pyrazol metabolite are potent inhibitors of the COX‐2 enzymatic activity in vitro and in vivo (Iñiguez et al., 2010; Wagemakers et al., 2009), while no COX‐2 inhibitory activity is attributed to the hydroxylated pyrazoline metabolite (manuscript under preparation). The proposed dose of enflicoxib to treat pain and inflammation associated with canine osteoarthritis is of 4 mg/kg administered once a week, with a first loading dose of 8 mg/kg (VMD, 2021).

In a pharmacokinetic study performed by Reinoso et al., (2001) in Beagle dogs, enflicoxib C_max_ was achieved between 4 and 8 h after its oral administration as unformulated drug and its pharmacokinetic profile after IV administration was characterised by a long elimination half‐life resulting from low plasma clearance and relatively large volume of distribution. Pharmacokinetic studies performed in rats by the same authors suggested that the long‐term efficacy of enflicoxib would be attributed to its active pyrazol metabolite (also described with its research acronym E‐6132), which shows a longer T_max_, longer terminal half‐life as compared to enflicoxib. However, pyrazol metabolite levels were not measured in the dog study and, hence, no information exists on its pharmacokinetic profile in dogs up to date.

In the present article, two pharmacokinetic studies assessing the behaviour of enflicoxib and its metabolites in Beagle dogs are presented. One is a pharmacokinetic study at single oral dose, in fed and fasted conditions to assess the effect of prandial state. In the same study, enflicoxib was administered at single intravenous dose to assess the absolute oral bioavailability, and the distribution into synovial fluid as the potential site of action was also investigated. The second study is a toxicokinetic evaluation in which the effect of different repeated oral doses in the plasma levels of enflicoxib and its pyrazol metabolite is described. Since drug efficacy may be affected by the degree of binding to the proteins within blood plasma, the in vitro assessment of the extent of protein binding of enflicoxib and its active metabolite in dog plasma is also included. In addition, the blood to plasma ratio of both compounds was also investigated in vitro.

## 2 MATERIALS AND METHODS

The studies followed parallel group study designs, because the expected long elimination half‐life of the pyrazol metabolite in the dogs implied an impractical washout period for crossover studies. They were conducted under the principles of Good Laboratory Practices (GLP), CFR (2019), OECD (1997) or UK Statutory Instrument (1999) No. 3106, as amended, and OECD (1997) Regulations and Directive 2004/10/EC (2004) of the European Parliament and of the Council. Both studies were reviewed and approved by the study site Institutional Animal Care and Use Committee and it followed current animal welfare recommendations NRC (2011), USDA (2013) or UK Animals (Scientific Procedures) Act (1986).

Study animals were non‐pregnant and non‐lactating and had not received NSAIDs or any other drugs within the month preceding the study. Dogs were subjected to clinical pathology evaluations and detailed veterinary examinations before being considered suitable for the studies.

### 2.1 Single dose oral and intravenous pharmacokinetic study

This study was designed to determine the oral bioavailability and the effect of food on plasma levels following oral administration, under the recommendations of current European pharmacokinetic guidelines (EMEA, 2000). The distribution into synovial fluid was prospectively investigated in samples corresponding to the last collection timepoint after the IV administration.

Thirty healthy Beagle dogs (males and females) of 1–5 years old at the time of dosing, and bodyweight between 7.4 and 12.3 kg, were housed indoors in climate‐controlled facilities in accordance with accepted laboratory animal care and use guidelines. As the animals belonged to the same centre, they were acclimatised for 5 days before study initiation.

Dogs were allocated in 4.6 m^2^ pens (with 5 animals per pen) into three treatment groups using the computer random number generator within the Microsoft^®^ Excel^®^ software. Animals were randomised to pens by sex, and treatment groups stratified by pre‐treatment bodyweight. Blocks were of size three, with each treatment occurring once within each block. Animals in the same block were housed in the same or neighbouring pens. Animals were moved to individual 0.75 m^2^ wire cages the evening prior to dosing to ensure fed or fasted conditions. Following the 24 h blood collection period, the animals were returned to their respective pens for the rest of the study.

Animals were kept at temperatures between 9 to 25°C and humidity from 32 to 90%. Artificial lighting was provided for approximately 12 h per day. Environmental enrichment was supplied to animals (e.g. toys).

Dogs were offered approximately 400 g of pelleted canine feed once daily. Fresh potable water was offered ad libitum. All dogs, except those in the Oral‐Fed group, were fasted overnight before dosing and food was offered after the 4‐h blood collection. Dogs in the Oral‐Fed group were supplied with 50% of their daily food ration approximately 1 h before administration, and the remainder of the food was given after the first 15‐min blood collection period post administration.

The oral groups received a single oral nominal dose of 8 mg/kg of enflicoxib (Daxocox^®^ tablets for dogs, Ecuphar/Animalcare) in its final formulation and were followed for 126 days. The dog’s mouth was opened, the dose placed towards the back of the tongue, and 5 ml of water was administered following the dose to aid in swallowing.

For the IV administration, a stable 2 mg/ml enflicoxib injectable solution was prepared in a vehicle containing 20% ethanol, 8% kolliphor HS‐15 and 72% water, and sterilised using a ≤0.22‐µm syringe filter. To avoid administering an excessive volume of product, dogs in the IV group received a single dose of 2 mg/kg and were followed for 35 days. A 22‐gauge ¾” catheter was introduced into the cephalic vein, using aseptic technique. A syringe with sterile saline was used to test the patency of the vein. The calculated volume of the solution was slowly administered through the catheter over approximately 1 min. Approximately 1–2 ml of sterile saline was used to flush the catheter following drug administration.

Blood samples (2 ml) were collected from the jugular vein in K_3_EDTA tubes for determination of plasma concentrations of enflicoxib and its metabolites, at the following time points:

Oral groups: Before administration (pre‐dose; Day 1 to Day 0), 15 and, 30 min, 1, 2, 4, 6, 8, 12, 24, 48 h and 3, 4, 6, 7, 10, 14, 21, 28, 42, 56, 70, 84, 98, 112, and 126 days post administration, with the aim to characterise the complete pharmacokinetic profile of the pyrazol metabolite.

IV group: Before administration (pre‐dose; Day 1 to Day 0), 2, 5, 10, 15 and 30m, 1, 2, 4, 6, 8, 12, 24 and 48 h and 3, 4, 6, 7, 10, 14, 21, 28 and 35 days post administration, to obtain reference values to calculate the oral bioavailability of enflicoxib. The hydroxylated pyrazoline metabolite was only determined in 130 selected plasma samples of the oral‐fed group corresponding to the timepoints pre‐dose, 0.5, 4, 8, 12, 24 and 48 h and 3, 6, 10, 14, 28 and 70 days post administration.

Blood samples were centrifuged within 30 min after collection at 2500 *g* for 10 min at *ca*. +5ºC. The resultant plasma was divided into 2 aliquots and frozen at *ca*. −20°C until analysis.

Samples of synovial fluid from the 10 dogs in the IV group were obtained under anaesthesia at 35 days post administration. Blank synovial fluid was obtained from 8 non‐dosed dogs at the same study facilities and pooled before being used for preparation of calibration curve and quality control samples for the qualification of the bioanalytical method.

### 2.2 Multiple dose oral toxicokinetic study

This study was designed as a Target Animal Safety (TAS) study, to assess the effects of repeated oral administrations of enflicoxib at either the recommended dose (1X), three times the dose (3X) or five times the dose (5X), that were compared to an untreated group (placebo, 0X) and followed the internationally agreed TAS guideline (VICH GL^43^, ^2008^). Blood samples were taken during the study to assess the dose‐exposure proportionality after single and repeated dose administrations. The toxicokinetic data from this study are discussed in this article, but safety aspects are reported elsewhere (Homedes et al., 2021).

Thirty‐two male and female healthy Beagle dogs, 8–10 months old and experimentally naïve, were obtained from a laboratory animal supplier. All animals were acclimatised for 28 days before study initiation. All dogs were housed in pairs of the same sex and dose group in indoor pens under temperature and humidity‐controlled conditions (15 to 24°C, 40–70% RH). Artificial lighting was provided for approximately 12 h per day. The pens were designed in accordance with the requirements of the UK Home Office Code of Practice for the Housing and Care of Animals (2014). For each group, periods of exercise and socialisation were permitted daily. Animals were fed daily with 400 g of a certified commercial pelleted diet. During the treatment period, food was offered immediately after dosing to maximise absorption according to the results of the single dose study.

Dogs were stratified by sex and weight, (16 males weighing 6.7–9.9 kg and 16 females weighing 5.3–7.0 kg) and allocated to four groups (0X, 1X, 3X and 5X) using a pseudo‐random body weight stratification procedure, which yielded groups of four dogs of each sex with approximately equal mean body weight. Dogs were distributed in a block of several adjacent pens and identified by a microchip inserted shortly after arrival.

Group 1X received the recommended dose of enflicoxib (8 mg/kg on the first day, as loading dose, and 4 mg/kg weekly thereafter, as maintenance dose); Group 3X received three times the recommended dose (24 mg/kg as loading dose and 12 mg/kg as maintenance dose); Group 5X received five times the recommended dose (40 mg/kg as loading dose and 20 mg/kg as maintenance dose) and Group 0X, received placebo tablets with similar appearance as the investigational drug at the same weekly intervals.

Enflicoxib was supplied as commercial Daxocox^®^ tablets containing 15, 30, 45, 70 or 100 mg of enflicoxib (Ecuphar/Animalcare). The number and size of the tablets administered orally were calculated weekly based on the most recent body weights and the target dose level required. Tablets were administered once weekly, for a total of 13 weeks (5X group) or 32 weeks (0X, 1X and 3X groups). Tablets administration was followed by 10–20 ml tap water to facilitate passage of drug to the stomach.

Blood samples were collected as described for the determination of blood concentrations of enflicoxib and its pyrazol metabolite. The sampling schedule was at 6 h (on week 1 only), 24, 48, 72, 96, 120, 144 and 168 h post dose in weeks 1, 13 and 31, Additionally, weekly samples were taken prior to each dose throughout the study. Plasma was obtained and stored as described before.

### 2.3 Analytical techniques

Plasma concentrations of enflicoxib and its metabolites were measured by two HPLC methods coupled with MS/MS detection (MRM) in negative ion electrospray mode. The methods had been previously validated according to current standards (EMA, 2009; FDA, 2018).

Method for enflicoxib and its pyrazol metabolite in plasma involved its extraction from 100 µL samples by solid phase extraction (Oasis MCX 30 μm 96‐well plates, 10 mg; Waters) and separated by reverse‐phase liquid chromatography on a Luna PFP (2) column (3 μm, 50 × 4.6 mm; Phenomenex), in gradient mode elution using 0.1% FA in water and MeOH 60:40 (v/v) as mobile phase flushed a flow rate of 1 ml/min. Celecoxib was used as internal standard.

Method for hydroxylated pyrazoline metabolite in plasma and for enflicoxib, pyrazol metabolite and hydroxylated pyrazoline metabolite in synovial fluid used the protein‐precipitation technique for extraction from dog samples (50 µl). The analytes were separated by reverse‐phase liquid chromatography on a Kinetex F5 100 Å column (100 × 3.0 mm; Phenomenex), in gradient mode elution using 0.1% FA in water and MeOH as mobile phase flushed a flow rate of 0.75 ml/min. Celecoxib was also used as internal standard.

The validation of the analytical methods demonstrated their selectivity, accuracy, precision, reproducibility and linearity. The concentration ranges were 5–1000 ng/ml for enflicoxib (2.5–600 ng/ml in synovial fluid), 2.5–1000 ng/ml for the pyrazol metabolite (2.5–600 ng/ml in synovial fluid) and 1–800 ng/ml for the hydroxylated pyrazoline metabolite (5–600 ng/ml in synovial fluid). The precision and accuracy of the respective determinations were always below 15% (below 20% for the lower limit of quantitation, ‐LLOQ) as per internationally accepted bioanalysis guidelines. Selectivity assessments demonstrated the absence of interfering peaks at the retention times of the analytes and the internal standard after analysis of drug‐free plasma or synovial fluid samples.

### 2.4 Pharmacokinetic analysis

The pharmacokinetic parameters were obtained from the individual plasma concentration‐time profiles of enflicoxib or its metabolites in each animal, by non‐compartmental analysis using Phoenix^®^ WinNonLin^®^ software (version 8.0. Pharsight Corporation). For all calculations, values below lower limit of quantification (BLLOQ) were considered as “0” except trailing concentrations, which were excluded from calculations.

For the single dose oral treatments, peak plasma concentration (C_max_) and time to peak concentration (T_max_) were reported. The apparent elimination rate constant λ_z_ was obtained by linear regression of the log‐linear terminal phase of concentration‐time profile using at least three data points, excluding T_max_ and calculated as the slope on the end points which maximise the R_sq_ adjusted ($r_{\text{adj}}^{2}$) coefficient (goodness of fit) requiring $r_{\text{adj}}^{2}$ > 0.80. Elimination or terminal half‐life (t_1/2_) was calculated by t_1/2_ = ln2 / λ_z_. The area under the concentration curve of plasma levels vs time from zero to the last quantifiable sample (AUC_last_) was calculated by the linear up/log down trapezoidal rule. The area under the concentration curve from zero to infinity (AUC_inf_) was the sum of AUC_last_ and the extrapolation after the last observed timepoint (AUC_extra_) that was calculated from the last quantified concentration divided by λ_z_. Absolute oral bioavailability and relative oral bioavailability (F% and F_rel_%) were calculated using actual doses between oral and IV administrations according to: F% = (AUC_inf_ oral * Dose IV )/(AUC_inf_ "IV *Dose oral)*100 and between oral‐fed and oral‐fasted groups according to the following formulation: F_rel_% = (AUC_inf_ oral fed *Dose fasted) / (AUC_inf_ oral fasted * Dose fed) * 100.

Additional pharmacokinetic parameters calculated for the IV treatment were plasma clearance (CL) as CL = Dose/AUC_inf_; volume of distribution at steady‐state (V_ss_) as V_ss_ = MRT_INF_ × CL; volume of distribution based on the terminal phase (V_z_) as V_z_ = CL/ λ_z_); and Mean resident time (MRT) was calculated as MRT = AUMC/AUC, with AUMC being the area under the first moment curve. For enflicoxib, C_0_ was calculated as the extrapolated plasma concentration at time zero after IV administration.

For the multi‐dose oral study, the pharmacokinetic parameters calculated for each dosing interval at weeks 1, 13 and 31 were the area under the plasma concentration‐time curve over the dosing interval (AUC_τ_ or AUC_168_), C_max_, T_max_ and the trough concentration at the end of the dosing interval (pre‐dose or C_min,ss_). The AUC_t_ and AUC_inf_ values were calculated with linear trapezoidal rule, and the extrapolation of AUC_t_ to obtain AUC_inf_ was performed using the observed concentration at the last time point. Due to the limited time between doses, the terminal rate constants (λ_z_), and corresponding terminal half‐lives (t_1/2_), were calculated only for the parent compound. The relationships between C_max_ and AUC_168_ of enflicoxib and its pyrazol metabolite and dose level were studied during weeks 1 and 13. The accumulation ratios, based on AUC_168_ values of both compounds, were calculated at weeks 13 (last experimental week for the 5X group) and 31 (last experimental week for the other groups).

For synovial fluid samples, the ratio of synovial fluid to plasma for enflicoxib and its metabolites was calculated at day 35 after IV administration.

### 2.5 In vitro plasma protein binding

Equilibrium dialysis studies were performed using the Rapid Equilibrium Dialysis (RED) devices (Waters et al., 2008) that consist of two chambers separated with a dialysis membrane with a cut‐off of 8.0 kDa (Pierce Biotechnology, ThermoFisher Scientific). The plasma protein binding was determined with blank canine plasma spiked with enflicoxib or its pyrazol metabolite. The highly bound compound tolterodine was used as a positive control. Stock solutions of the tested compounds were prepared at 10 mM concentration in DMSO and were immediately used or stored at −20 °C (±5°C) until further use.

Samples were prepared by spiking dog plasma with the compounds at three increasing concentrations (0.4 µM, 1 µM and 4 µM). Plasma samples were loaded (200 µl) in the donor chambers while the opposite chamber contained 350 µl of dialysis buffer (0.1 M phosphate buffer, pH 7.4). Dialysis was run at 37°C for 4 h with continuous shaking. Samples (50 µL) were harvested at t_0_ and after 4 h of incubation from both chambers and brought up to 100 µL either with plasma or dialysis buffer to reach the same proportion of plasma in all samples (matrix matching). Dialysis of test and reference compounds in the absence of plasma (chambers filled with dialysis buffer) were used to assess non‐specific binding. Studies were also performed to estimate recovery from the dialysis system. The unbound fractions for enflicoxib or its pyrazol metabolite were determined by LC‐MS/MS using a modification of the previously described method. Standard calibration curves ranged from 0.005 µM to 8 µM for test and reference compounds were prepared in dog plasma‐matched matrix and treated as the test samples.

The percent of binding to plasma proteins was calculated as:

$$\%{\text{Protein binding = (C}}_{\text{donor}}‐C_{\text{acceptor}})/C_{\text{donor}\;}*100.$$

where: C_donor_ = Concentration in the donor chamber after dialysis; C_acceptor_ = Concentration in the acceptor chamber after dialysis. The value of C_donor_ (0) = Concentration in the donor chamber prior to dialysis, was used to determine the recovery of the assay.

For each plasma sample, protein binding was determined in triplicate.

### 2.6 In vitro blood to plasma partitioning

Whole blood was obtained from 3 untreated female Beagle dogs (located in Isoquimen SL, Sant Feliu de Codines, Barcelona, Spain) and pooled into two different batches. Lithium heparin was used as anticoagulant. Blood was maintained in a rotator shaker at room temperature before use. Reference plasma was obtained from the same whole blood pool by centrifugation at 2000 × *g* for 15 min at *ca* 4°C.

Stock solutions of the test compounds enflicoxib and pyrazol metabolite, the positive control chloroquine and the internal standards celecoxib and chloroquine‐d4, were stored at *ca* −20°C. The derived working solutions were freshly prepared each day of experimentation.

The haematocrit (H), percent of total blood cells in whole blood samples (v/v), was determined before the assay. Blood was spiked with enflicoxib and its pyrazol metabolite at three increasing concentrations (1 µg/ml, 5 µg/ml and 15 µg/ml) and with 500 nM chloroquine. The samples were incubated separately (*n* = 3) at *ca* 37°C for 60 min under gentle agitation in a rotator shaker in polypropylene tubes. The concentration of organic solvent in the incubates was kept below 0.05% to prevent haemolysis. Reference plasma was incubated similarly in Eppendorf protein LoBind tubes as a surrogate matrix of whole blood to assess total drug concentration (C_b_). After incubation, the blood samples were centrifuged at 2000 × *g* for 15 min at *ca* 4°C and the resulting plasma was stored frozen together with the reference plasma (*ca* −80°C) in 100 µl or 50‐µl aliquots.

For the enflicoxib and pyrazol metabolite determination, the plasma specimens were mixed with AcN containing the internal standard celecoxib for protein precipitation (1:3 ratio plasma: solution). The samples were vortexed and subsequently centrifuged at 5000 *g* for 15 min. After centrifugation, the supernatants were diluted with 0.1% FA in water: AcN (90:10) and injected into the LC‐MS/MS system. For chloroquine measurement, plasma specimens were processed similarly. Blank samples were processed as described before, and were used to check the selectivity and carry‐over of the LC‐MS/MS analytical runs.

The quantification of each test item in plasma was performed using calibration curves ranging from 0.05 to 20 µg/ml. For chloroquine quantification, the calibration curve samples were prepared similarly at plasma concentrations from 50 to 2000 nM. In both cases, the LC‐MS/MS response ratios (peak area of analyte/peak area Internal Standard) were used for quantitation.

The analyte concentration obtained from LC‐MS/MS analysis in each sample was used to calculate the blood‐plasma concentration ratio (*C*_b_/*C*_p_) as follows:

*C*_b_/*C*_p_ = [(concentration in reference plasma; Equivalent to concentration in whole blood) / (concentration in plasma from whole blood)].

From these values, the red blood cells (RBC) to plasma ratio (*K*_RBC/PL_) was also calculated using the following equation:

$$K_{\text{RBC}/\text{PL}}=1/H\times \left(\frac{C_{b}}{C_{p}}‐1\right)+1$$

where H: haematocrit.

The mean ± SD for the values obtained in the replicates for each species evaluated were calculated.

### 2.7 Statistical analysis

For the single dose study, descriptive statistics for PK parameters were calculated per treatment. The data were logarithmically transformed prior to analysis based upon fundamental pharmacokinetic relationships (Steinijans & Hauschke, 1990). C_max_ and AUC values were subject to analysis of variance (ANOVA) including terms for animals, sex and feeding condition (i.e. fed or fasted). The 90% confidence interval for the difference of fed and fasted was constructed using the error variance obtained from the ANOVA. The interval estimated was then back transformed to give estimates of the ratio of fed relative to fasted. AUC and C_max_ were compared using a mixed effects model analysis. The alpha risk was set at 0.05. Fixed effects were fed and fasted conditions.

A significant food effect was established if the 90% confidence intervals for C_max_ and AUC was out of the pre‐specified range of 0.80–1.25.

The influence of sex was evaluated on C_max_ and AUC using T‐test for the comparison of means using Statgraphics 5.1 (Statgraphics Technologies, Inc). A statistically significant effect was determined when the *p* value was <.05.

For the repeated oral dose study, analyses of variance were performed for week 1 and week 13 data separately, including all three dose levels to assess dose proportionality. For weeks 13 and 31, the analysis was done for the low and mid dose levels only, adjusted to 1 mg/kg. The factors in these analyses were dose group, time, sex and their interactions. Animal was included as a blocking factor since the same animals were bled on each sampling day. The data were logarithmically transformed prior to its analysis using SAS 9.4 (SAS Institute (2002) SAS OnlineDoc^®^ Version Nine. SAS Institute Inc.).

For synovial fluid concentrations, plasma protein binding and blood to plasma partitioning, descriptive statistics were calculated.

## 3 RESULTS

### 3.1 Single‐dose pharmacokinetic study

**FIGURE 2:**
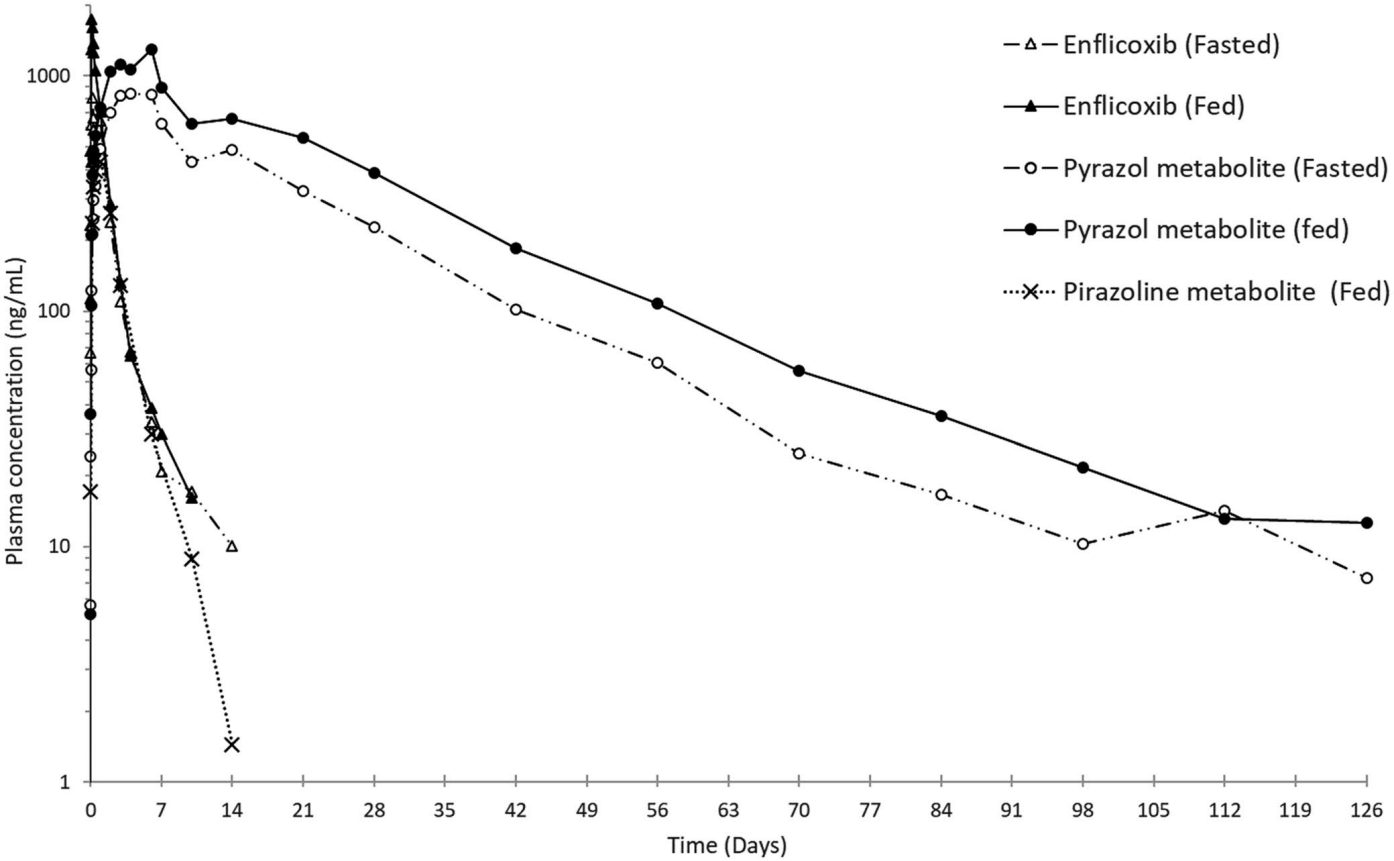
Graphical representation of average blood levels of enflicoxib and its metabolites after a single oral dose administration of 8 mg/kg of enflicoxib in fed or fasted conditions. Semi‐logarithmic scale

**FIGURE 3:**
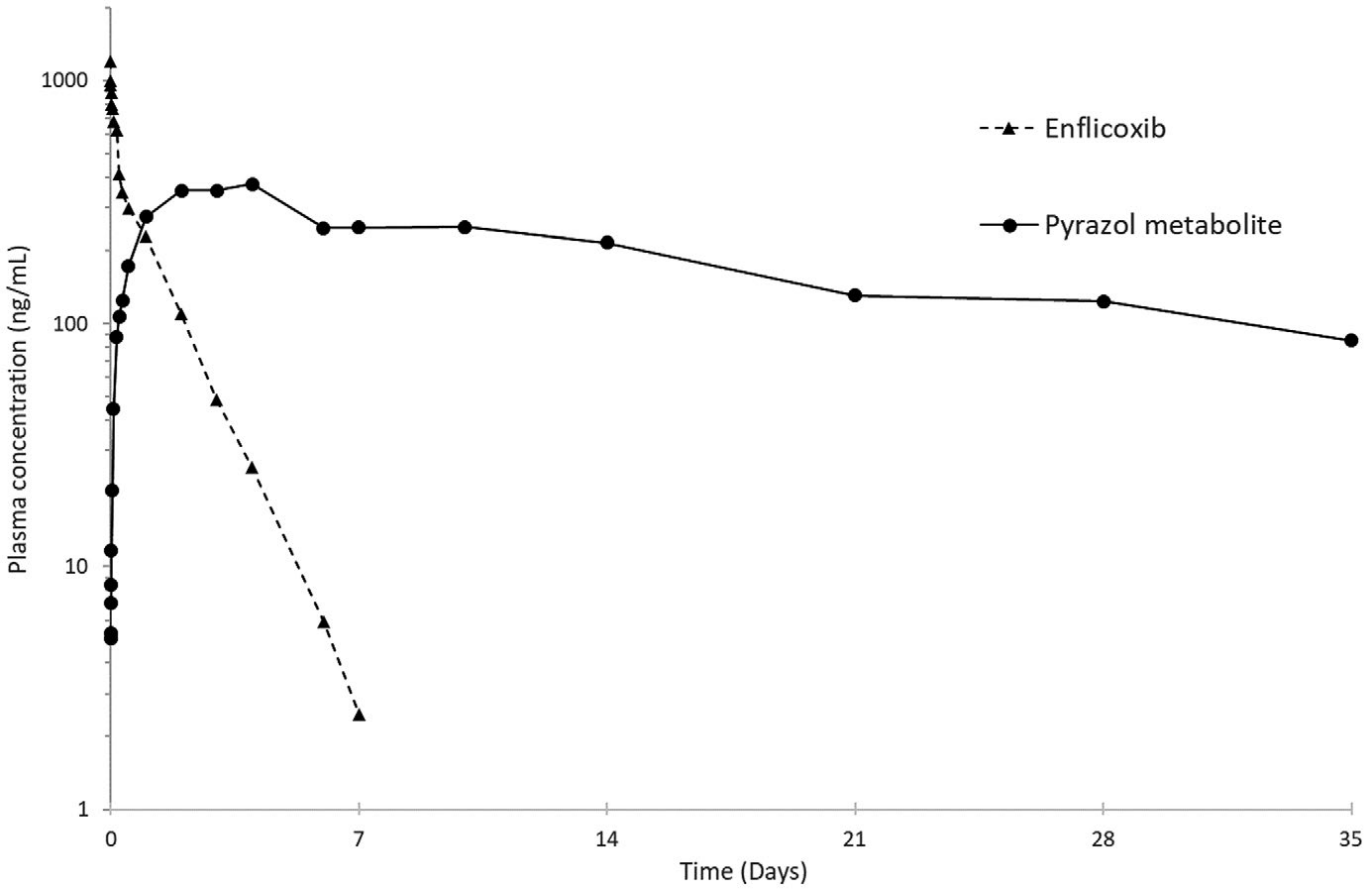
Graphical representation of average blood levels of enflicoxib and its pyrazol metabolite after a single intravenous dose administration of 2 mg/kg of enflicoxib. Semi‐logarithmic scale

The pharmacokinetic parameters of enflicoxib and its metabolites after oral administration of 8 mg/kg of enflicoxib to fed or fasted dogs are summarised in Table 1. The pharmacokinetic parameters after intravenous administration of 2 mg/kg of enflicoxib are summarised in Table 2. Drug concentrations versus time profiles after single oral doses of 8 mg/kg (fed and fasted) and 2 mg/kg IV are shown in Figure 2 and Figure 3.

**TABLE 1:** Pharmacokinetic parameters of enflicoxib and its metabolites after oral administration of enflicoxib to dogs in fed or fasted conditions (*n* = 10 dogs/group)

Group	Enflicoxib		Pyrazol metabolite		OH‐Pyrazoline metabolite
	Fed	Fasted	Fed	Fasted	Fed
C_max_ * (ng/ml)	1857 ± 415.7 (22.4)	1028 ± 644.9 (62.8)	1311 ± 237.2 (18.1)	890.3 ± 551.3 (61.9)	447 ± 101 (22.5)
T_max_ ^†^ (h)	2.0 [1.0–4.0]	4.0 [1.0–24.0]	144.00 [48.00–144.03]	120.00 [48.00–336.00]	24 [12.0–24.0]
t_1/2_ ^‡^ (h)	19.68 [13.4–35.8]	18.33 [10.6–59.2]	406.1 [321–658]	353.9 [255–491]	42.7 [34.4–55.9]
AUC_last_ * (ng·h/ml)	46,560 ± 18,860 (40.5)	31,300 ± 22,710 (72.6)	6,62,000 ± 1,68,500 (25.4)	3,91,500 ± 2,55,100 (65.2)	27,578 ± 5592^$^ (20.3)
AUC_inf_ * (ng·h/ml)	47,010 ± 18,980 (40.4)	31,720 ± 22,740 (71.7)	6,69,300 ± 1,73,100 (25.9)	4,22,700 ± 2,54,500 (60.2)	
F%	63.1	44.1	75.4	49.3	
F_rel_%	143.3	‐	153.1	‐	

* Mean ± SD (CV%).

† Median [min‐max].

‡ Harmonic mean.

$ T_last_ > 240 h.

**TABLE 2:** Pharmacokinetic parameters of enflicoxib and its pyrazol metabolite after intravenous administration of 2 mg/kg of enflicoxib to 10 dogs

Group	Enflicoxib	Pyrazol metabolite
C_max_ * (ng/ml)	‐	384.5 ± 123.8 (32.2)
T_max_ ^†^ (h)	‐	96 [48–96]
t_1/2_ ^‡^ (h)	19.77 [11.2–29.7]	335.6^$^ [325.8–347.0]
AUC_last_ * (ng·h/ml)	16,190 ± 5707 (35.3)	1,56,500 ± 39,810 (25.4)
AUC_inf_ * (ng·h/ml)	16,520 ± 5806 (35.1)	1,96,900 ± 58,570^$^ (29.7)
C_0_^1^ (ng/ml)	1353 ± 420.2 (31.1)	‐
V_Z_ * (ml/kg)	3995 ± 965 (24.2)	‐
Cl* (ml/h/kg)	140.2 ± 63.92 (45.6)	‐
MRT_inf_ * (h)	29.22 ± 9.29 (31.8)	‐
MRT_last_ * (h)	‐	329.5 ± 27.46 (8.3)

Abbreviation: PE, point estimate.

* Mean ± SD (CV%).

^†^ Median [min‐max].

^‡^ Harmonic mean [min‐max].

^$^ *n* = 4.

After oral administration, the bioavailability of enflicoxib is 44.1% in fasted conditions and 63.4% in fed conditions and could be classified as moderate (Nomeir et al., 2009). Enflicoxib is rapidly metabolised as shown by the first concentrations of both metabolites detected at the first sampling time after dosing. Enflicoxib plasma concentrations were higher than those observed for the metabolites during the first 24–48 h after administration, whereas plasma levels of the pyrazol metabolite were 12‐fold higher than those observed for enflicoxib from 48 h to the end of the study. The rate of absorption was slightly higher for the oral route in fed conditions. The C_max_ was reached at 2 h in fed conditions and at 4 h in fasted conditions, although this different rate was not detected in the pyrazol metabolite formation. Food also increased by 43% the extent of absorption and the plasma exposure. The elimination of enflicoxib (t_1/2_) was 20 h after IV or oral administration whatever the feeding condition.

The pharmacokinetic profile for the pyrazol metabolite was different from that for enflicoxib with long formation and elimination phases. T_max_, was shorter after the IV administration (median 96 h versus 120–144 h for oral doses), but similar t_1/2_ values were obtained in all routes of administration (ranging from 336 to 406 h). After the IV administration, the elimination phase of this metabolite was not always well defined due to the short sampling period compared to its slow elimination rate. Consequently, only data from 2 males and 2 females were considered for the mean calculation of t_1/2_, V_z_, CL and AUC_inf_. MRT values were much lower for the pyrazol metabolite than for enflicoxib.

The hydroxylated pyrazoline metabolite had a faster formation rate (T_max_ 24 h) than pyrazol metabolite, but its concentrations were always much lower than enflicoxib or the pyrazol metabolite and declined at an intermediate rate, with an average t_1/2_ of 43 h.

As occurred in plasma, at day 35 after the IV administration, the levels of enflicoxib were BLLOQ in all synovial fluid samples. Therefore, its ratio versus plasma concentration could not be calculated. The pyrazol metabolite showed values between 2.8 ng/ml and 3.3 ng/ml and its ratio versus plasma at 35 days’ post dose ranged from 0.031 to 0.078 (3.1% to 7.8%).

### 3.2 Multiple dose pharmacokinetic study

Enflicoxib at all three dose levels was well tolerated by all dogs. The actual measured mean dose levels for the groups 1X, 3X and 5X were 5.1, 12.0 and 20.1 mg/kg/week, respectively. Individual enflicoxib doses exceeded the intended oral dose levels particularly in the therapeutic dose group, in which all animals received the highest dose according to tablet size distribution by weight.

**FIGURE 4:**
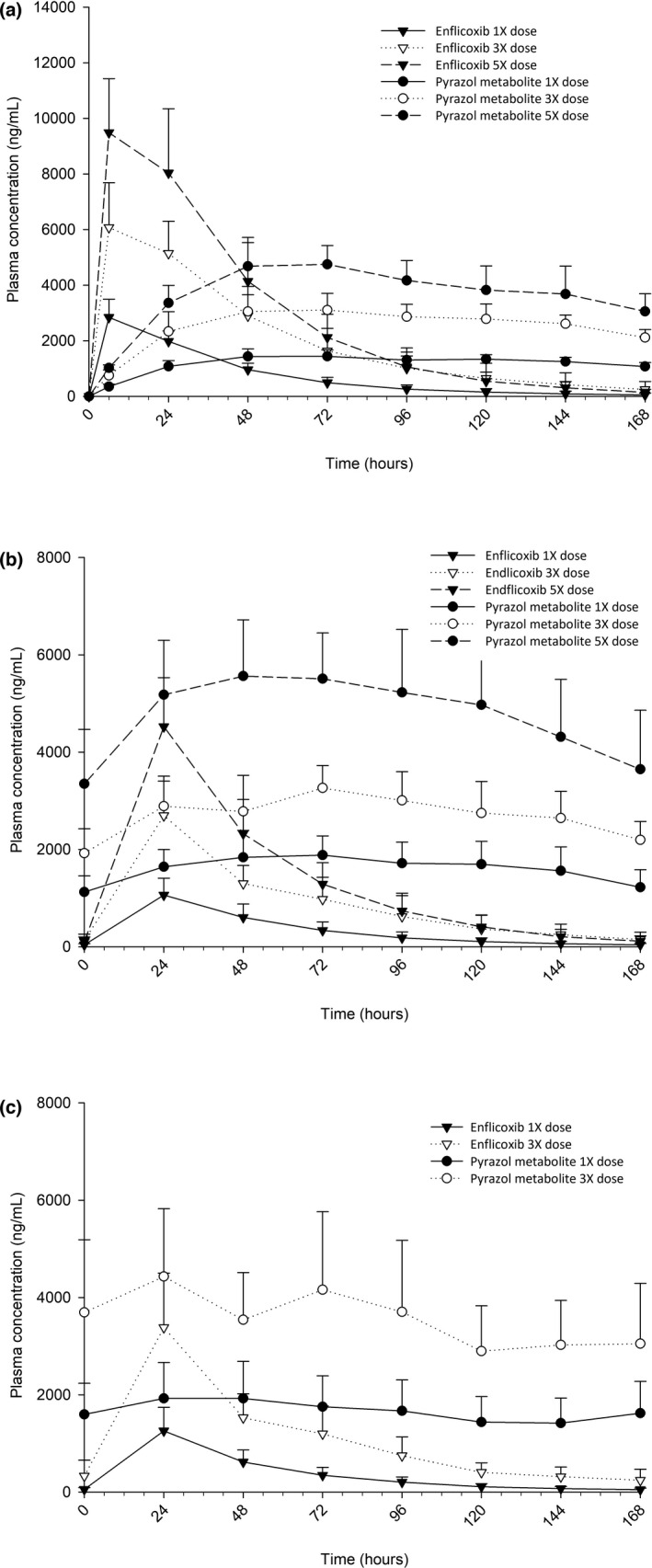
Graphical representation of blood levels of enflicoxib and its pyrazol metabolite during weeks 1 (a), 13 (b) and 31 (c) in healthy Beagle dogs treated with enflicoxib orally at a loading dose and once‐a‐week nominal doses of 8 + 4 mg/kg (1X), 12 + 24 mg/kg (3X) and 40 + 20 mg/kg (5X) (mean ± SD)

The pharmacokinetic profiles during weeks 1, 13 and 31 of treatment are depicted in Figure 4. After the initial loading dose administration on week 1, the plasma concentrations of enflicoxib increased at a similar rate reaching its C_max_ after 6 h (first sampling time) in all groups in a dose‐proportional manner, and returned to near baseline levels by the end of the week (168 h). The pyrazol metabolite levels reached its C_max_ at 72 h (48–120 h), regardless of the dose, and decreased slowly maintaining high levels up to the last sampling at 168 h.

The behaviour of enflicoxib on week 13 was very similar to week 1, as pre‐dose concentrations were very low in all groups (below 100 ng/ml). After reaching the peak concentrations during the first day, plasma levels returned to the pre‐dose concentrations by the end of the week. On the other hand, pyrazol metabolite pre‐dose levels were high (above 1000 ng/ml), but proportional to the actual dose administered in the previous weeks. Metabolite C_max_ was reached at an earlier time compared to the initial loading dose on week 1 (24 vs. 72 h), and plasma concentrations decreased slowly thereafter reaching the same pre‐dose levels by the end of the week.

Plasma profiles of enflicoxib on week 31 (only groups 1X and 3X) were like those for week 13. Pyrazol metabolite levels were somewhat higher in the 3X group, but in the 1X group remained at a similar level compared to week 13.

**FIGURE 5:**
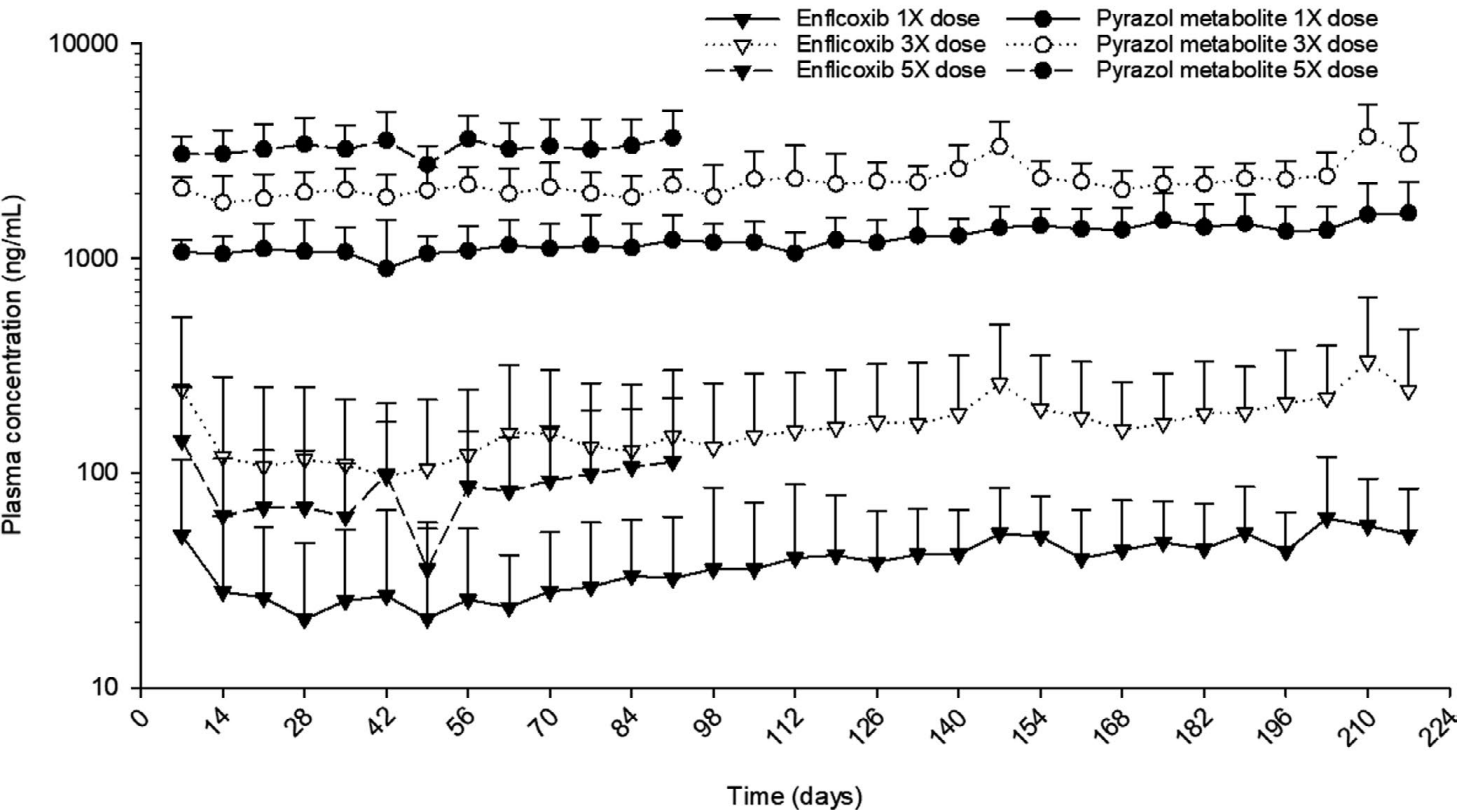
Graphical representation of pre‐dose blood levels of enflicoxib and its pyrazol metabolite in healthy Beagle dogs treated with enflicoxib for 7 months orally at a loading dose and once‐a‐week nominal doses of 8+4mg/kg (1X), 12 + 24 mg/kg (3X) and for 3 months at 40 + 20 mg/kg (5X) (mean ± SD)

Pre‐dose plasma concentrations (C_min,ss_) throughout the study are illustrated in Figure 5. Enflicoxib plasma concentrations were higher after the administration of the loading dose, but subsequently remained very constant at very low levels, regardless of the treatment group. Pre‐dose plasma concentrations were highest in the animals in the 3X group, which probably resulted from inter‐animal variation as the systemic exposure, generally increased with increasing dose. Pre‐dose concentrations of the pyrazol metabolite were high after the loading dose administration and remained constant or slightly increased thereafter at concentrations proportionate to the dose administered. No trend to any significant over‐accumulation was seen at any dose level.

The rate of absorption (C_max_) and extent of systemic exposure (AUC_168_) to enflicoxib in dogs increased proportionately with increasing dose over the dose range 8/4 to 40/20 mg/kg/week during weeks 1 and 13 (See Table 3). No differences were found between sexes (data not shown). Taking into account the mean actual doses administered (11.3/5.1; 24.4/11.9 and 39.6/20.1 mg/kg for the 1X, 3X and 5X groups, respectively), the relationships between the adjusted PK parameters and the dose level followed a linear pattern with correlation coefficients higher than 0.8 for enflicoxib and 0.9 for the metabolite (See supplementary material). On week 31, similar behaviour is seen between the 1X and 3X doses (data not shown).

**TABLE 3:** Pharmacokinetic parameters (mean ± SD) of enflicoxib or its pyrazol metabolite during weeks 1, 13 and 31 in healthy Beagle dogs treated with enflicoxib orally at a loading dose and once‐a‐week nominal doses of 8 + 4 mg/kg (1X), 12 + 24 mg/kg (3X) and 40 + 20 mg/kg (5X) (*n* = 8 dogs/group)

Group	C_max_ (ng/ml)					
	Week 1		Week 13		Week 31	
	Enflicoxib	Pyrazol metabolite	Enflicoxib	Pyrazol metabolite	Enflicoxib	Pyrazol metabolite
1X	2021 ± 461	1087 ± 215	878 ± 319	1316 ± 263	1454 ± 374	2111 ± 561
3X	6051 ± 1394	3257 ± 457	2760 ± 817	2955 ± 537	3384 ± 1117	4645 ± 1360
5X	9587 ± 2042	4904 ± 775	4390 ± 1052	4874 ± 925	‐	‐

Group	AUC_168_ (ng·h/ml)					
	Week 1		Week 13		Week 31	
	Enflicoxib	Pyrazol metabolite	Enflicoxib	Pyrazol metabolite	Enflicoxib	Pyrazol metabolite
1X	87,943 ± 21,461	1,43,833 ± 20,920	47,649 ± 23,028	2,19,721 ± 47,895	63,974 ± 26,133	2,81,925 ± 1,04,711
3X	3,35,052 ± 1,08,913	4,23,179 ± 70,000	1,57,215 ± 63,585	4,70,026 ± 89,761	1,88,745 ± 69,379	6,03,475 ± 1,84,027
5X	4,85,509 ± 1,56,853	6,32,268 ± 1,09,131	2,16,547 ± 63,957	76,8,738 ± 1,43,248	‐	‐

After repeated weekly doses, the rate of absorption (C_max_) and extent of systemic exposure (AUC_168_) to enflicoxib in dogs were lower than those values obtained after the initial loading dose administration on week 1, as the maintenance dose was half of the initial dose. The accumulation ratios, based on AUC_168_ values, were less than one (approximately 0.5 for all three dose levels on week 13 and week 31) indicating that systemic exposure to enflicoxib after repeated weekly administration was lower than after the administration of the loading dose during all study period, demonstrating absence of time‐dependency. When normalising the loading dose with the dose value of maintenance dose, the accumulation ratios of enflicoxib were close to 1, and for the pyrazol metabolite ranged from 2.1 to 3.2. However, when comparing week 13 and week 31, the accumulation ratio was around 1.3 at both dose levels, confirming that levels are close to steady state at week 13 without over‐accumulation and suggests that the steady state is almost reached on week 13 (see Table 4).

**TABLE 4:** Normalised accumulation ratios of enflicoxib and its pyrazol metabolite during weeks 13 and 31 vs Week 1 in healthy Beagle dogs treated with enflicoxib orally at a loading dose and once‐a‐week nominal doses of 8 + 4 mg/kg (1X), 12 + 24 mg/kg (3X) and 40 + 20 mg/kg (5X) (*n* = 8 dogs/group)

Group	Enflicoxib		Pyrazol metabolite	
	Week 13	Week 31	Week 13	Week 31
1X	0.93	1.05	2.78	2.82
3X	0.9	1.15	2.21	2.86
5X	1	‐	2.67	‐

### 3.3 In vitro plasma protein binding

After the 4‐h incubation time, the plasma protein binding of enflicoxib and its pyrazol metabolite was independent of the concentration at the assayed levels of 160, 400 and 1600 ng⁄ml, with mean binding percentage values ranging from 97.38% to 99.01% for enflicoxib and from 99.35 to 99.72% for the metabolite. Values for the reference standard tolterodine were as expected, thus validating the assays. Non‐specific binding was found irrelevant since recovery was close to 100% for the metabolite.

### 3.4 In vitro blood to plasma partitioning

**FIGURE 6:**
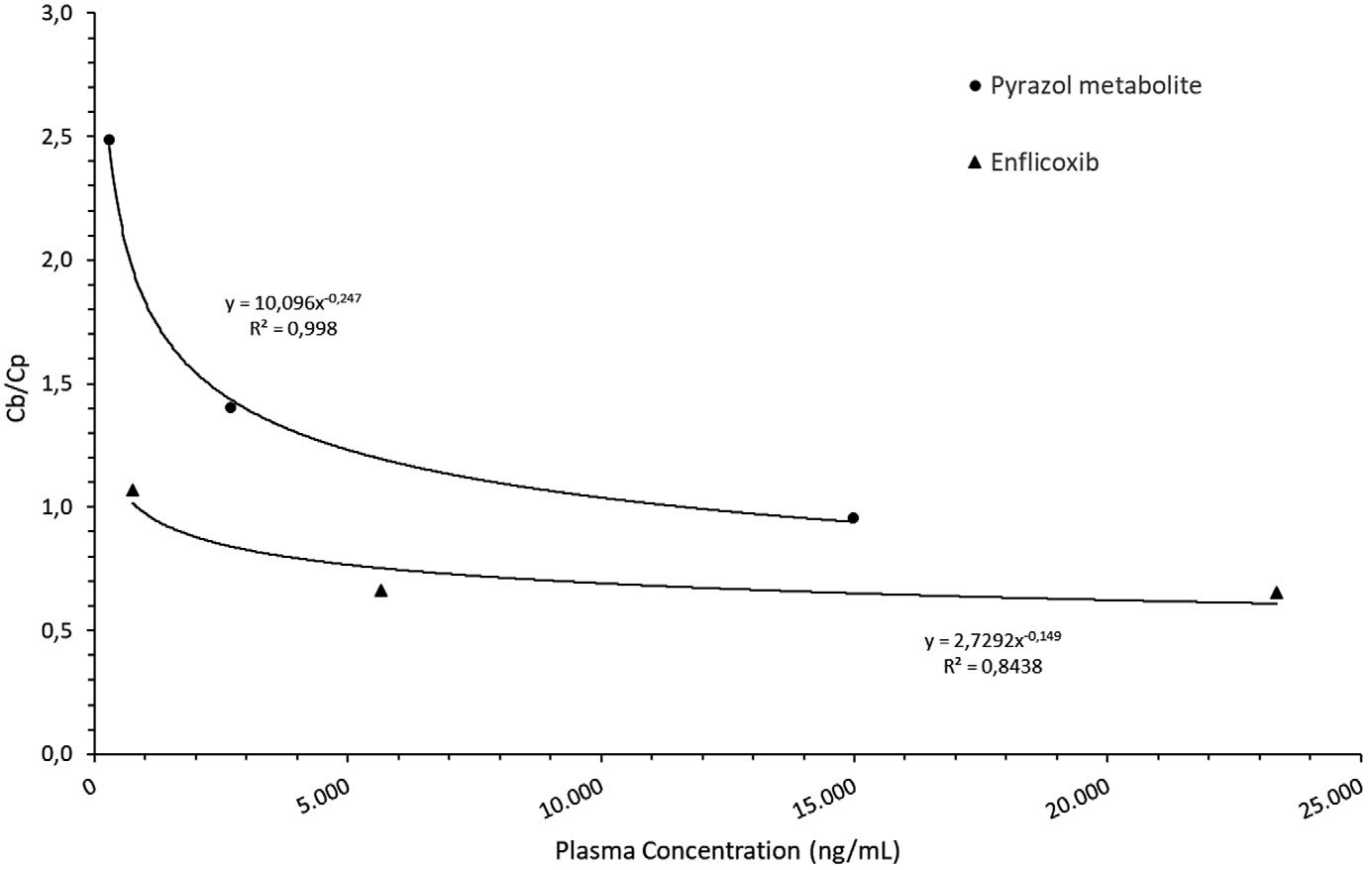
Partitioning of enflicoxib and its pyrazol metabolite in dog blood and plasma at increasing concentrations

Enflicoxib showed higher affinity for the plasma compartment, particularly at the high and medium blood concentrations (15 µg/ml and 5 µg/ml, Cb/Cp: 0.66), while it was equally distributed between red blood cells and plasma at the lowest concentration tested 1 µg/ml (Cb/Cp: 1.07). For the pyrazol metabolite, the partitioning was favourable to the cell compartment and was also dose dependent. The ratio Cb/Cp was found to increase from 0.95 to 1.40 and 2.49 at the blood concentrations of 15 µg/ml, 5 µg/ml and 1 µg/ml, respectively. Therefore, the blood to plasma ratio showed saturable kinetics for both compounds, with higher blood cell affinity at lower total blood concentrations (See Figure 6). Values for the reference standard chloroquine were as expected, showing high affinity for the blood cell compartment (data not shown), thus validating the assays.

## 4 DISCUSSION

The results of the studies in the present work confirm that, after its oral administration to Beagle dogs, the pharmacokinetics of enflicoxib are characterised by its slow absorption and relatively long elimination half‐life. After absorption, enflicoxib is metabolised forming two main metabolites corresponding to a hydroxylated pyrazoline and a pyrazol derivative. When comparing with the parent compound enflicoxib, the hydroxylated pyrazoline metabolite shows lower plasma levels, but a similar pharmacokinetic profile, while the pyrazol metabolite reaches lower peak levels, but shows a much slower elimination. The long persistence of the pyrazol metabolite in plasma makes crossover study designs unfeasible. Therefore, parallel group designs were inevitably used in these studies, despite implying a larger interindividual variability compared to crossover designs.

After a single oral dose administration, the rate of absorption for enflicoxib was higher in fed than in fasted conditions, although this did not translate to increase the rate of formation of the pyrazol metabolite, which reached its C_max_ at the same time whatever the fed/fasted state. A higher variability was observed for enflicoxib in comparison with the pyrazol metabolite, mainly during terminal phase. The slow elimination rate of the metabolite compared to the parent compound suggests that its decline is controlled by its elimination half‐life, and not governed by the elimination of the parent compound. The increased affinity of the pyrazol metabolite at low concentrations towards red blood cells, together with its higher extent of binding to plasma proteins, is likely to also contribute to its slow elimination rate. The long terminal half‐life of the metabolite explains its persistent systemic availability, and confirms the findings described in rats (Reinoso et al., 2001) as being responsible for the long‐term efficacy after the administration of enflicoxib. On the other hand, the large variability in T_max_ for the pyrazol metabolite (48–144 h and 48–336 h for the fed and fasted conditions, respectively) could be explained by its relatively flat concentration‐time profile during the first week after dosing. The reduced frequency of blood sampling after the 24 h post‐dose could have also contributed to a diminished ability to detect T_max_ more accurately.

After IV administration, enflicoxib concentrations declined quite homogeneously, with an average t_1/2_ of 20 h, similar to the oral administrations. The C_max_ for the pyrazol metabolite was achieved somewhat earlier than in the oral routes (48–96 h post administration).

The oral bioavailability also increased when enflicoxib was given with food, as has been described for other NSAIDs like celecoxib, mavacoxib or vitacoxib (Cox et al., 2010; Paulson et al., 2001; Wang et al., 2019). When enflicoxib was administered with food, the oral bioavailability was higher than in fasted conditions (63 vs 44%, respectively), indicating that the drug has relatively good permeability, as would be suggested by its V_z_ values. Enflicoxib is practically insoluble in water (Ecuphar/Animalcare internal data), and the increase in bioavailability by food could be consistent with enflicoxib being a poorly water‐soluble/high‐permeability drug. Food could increase the bioavailability by increasing gastric residence time to allow more time for tablet dispersion and drug dissolution, better dissolution in the fat content of dog’s diet, or by facilitating dissolution and increasing solubility via meal‐stimulated secretion of bile salts. Due to its higher bioavailability in fed conditions, the repeated dose study was performed administering the product with food, and this is the method of administration recommended for the commercial product Daxocox® (VMD, 2021). The long‐term exposure of the pyrazol metabolite could be attributed to its slow formation from the parent compound and its long terminal half‐life. This would suggest a long‐term exposure to the metabolite in the target tissue. In this study, levels of pyrazol metabolite in the joints as a target site of action, as measured in the synovial fluid, were of some 10% of those in plasma, and thus no tissue accumulation is evidenced.

Terminal half‐life values of enflicoxib are similar to other NSAIDs approved for daily use in dogs like carprofen, firocoxib, robenacoxib or cimicoxib (Jeunesse et al., 2013; Jung et al., 2009; McCann et al., 2004; Schmitt & Guentert, 1990). However, the pyrazol metabolite shows much longer terminal half‐life than enflicoxib, which is compatible with a weekly dosing schedule. A longer terminal half‐life has been described in the other long‐acting authorised product, mavacoxib which also allows for extended dosing intervals (Cox et al., 2010).

Enflicoxib exhibits relatively large between‐subject variability in its elimination parameters, particularly on t_1/2_, which spanned a 3‐ to 6‐fold range of values. However, when the pyrazol metabolite is considered, a much homogeneous and narrow range of 1‐ to 2‐fold was achieved. Despite the limited population of this single dose study, no subgroups of slow metabolisers can be identified, particularly for the pyrazol metabolite, as it has been described in similar studies with Beagle dogs for other NSAIDs such as celecoxib (Paulson et al., 2001), cimicoxib (Jeunesse et al., 2013) or mavacoxib (Cox et al., 2010, 2011). This homogeneous elimination pattern of the more persistent pyrazol metabolite indicates a predictable behaviour when a repeated chronic treatment is envisaged.

Because of the relatively long t_1/2_ of the pyrazol metabolite, a regimen with a loading dose has been used as a strategy to attain rapidly steady‐state concentrations, which are later maintained relatively constant by weekly maintenance doses. The studies used for the dose determination in animal models and to demonstrate the therapeutic efficacy of this dosage for the treatment of canine osteoarthritis are described elsewhere.

The dose‐normalised parameters both for enflicoxib and for the pyrazol metabolite were studied during an entire dosing interval (168 h) after the first loading dose administration and at weeks 13 and 31 after several maintenance weekly doses. The hydroxylated pyrazoline metabolite was not measured during this study, as it has been recently demonstrated that it lacks COX‐1 or −2 inhibitory activity (manuscript under preparation) and therefore considered not relevant from the efficacy or safety point of view. As expected, similar C_max_ values as in the previous single dose study were obtained during the first week. The C_max_ and AUC_168_ of both compounds showed dose proportionality at single loading doses from 8 to 40 mg/kg in the first week but also after repeated weekly administrations at maintenance doses from 4 to 20 mg/kg. Accumulation ratios were less than 1 for the parent compound and between 2.1 and 3.2 for the metabolite as compared to the initial loading dose (normalised).

However, comparisons between weeks 13 and 31, where the same maintenance dose is administered, seem more meaningful in terms of estimation of potential accumulation for the pyrazol metabolite in long‐term treatments. There was no change in systemic exposure to parent compound between weeks 13 and 31, particularly at the lowest dose.

Systemic exposure to the pyrazol metabolite in weeks 13 and 31 was somewhat higher than that after administration of the initial loading dose in week 1. However, the mean AUC_168_ values were similar during weeks 13 and 31, with accumulation ratios during this period ranging from 0.56 to 1.49 in the therapeutic dose group, indicating that there was no significant change in systemic exposure during this period, in any dog. Likewise, after the first week, trough plasma concentrations were relatively constant and homogeneous up to the end of the experimental period (3 months for the highest dose group and 7 months for the low and intermediate dose groups), thus confirming the lack of over‐accumulation in any dog. These stable long‐term concentrations and the predictable elimination pattern of the pyrazol metabolite would support the adequacy of the weekly dose interval proposed.

Like most NSAIDs, enflicoxib and its pyrazol metabolite are highly bound to plasma proteins, which would limit its tissue penetration and elimination rate (Páhlman & Gozzi, 1999; Scheife, 1989). This seems to be confirmed in these studies according to the low concentrations found in the synovial fluid and the low terminal half‐life obtained for the pyrazol metabolite. Also, the blood to plasma ratio provides an indication of drug binding to erythrocytes. Overall, the blood partitioning of enflicoxib and its pyrazol metabolite showed a saturable kinetics, as enflicoxib was mainly distributed to the plasma compartment at high blood concentrations, while it was equally distributed between red blood cells and plasma at 1 µg/ml (Cb/Cp: 1.07). On the other hand, the pyrazol metabolite showed higher affinity than the parent compound for the blood cells, especially at low concentrations. An enhanced uptake of the pyrazol metabolite into the erythrocytes could be one possible explanation for its longer in vivo half‐life observed as compared to the parent drug. The kinetics of the blood cells distribution of enflicoxib and the metabolite could possibly induce a decrease in the plasma CL of both compounds due to a potential “depot” effect exerted by the high affinity to the red blood cells, which decreases the availability of the compounds to the enzymes. This finding may be more meaningful in the case of the pyrazol metabolite.

In conclusion, the rate and extent of systemic exposure of dogs to enflicoxib and its pyrazol metabolite is characterised by dose‐independent (linear) kinetics and the prolonged half‐life for the metabolite supports its proposed dosing interval in which doses are separated by one week. Sustained levels of active in blood, and therefore of prolonged COX‐2 inhibition, combined with a low frequency dose interval would be very beneficial in terms of efficacy and treatment compliance by owners (EMEA, 2008; Lees et al., 2015) and offer more stable levels that would achieve a more constant therapeutic response.

## CONFLICT OF INTEREST

JH and MS are employees of Ecuphar veterinaria S.L.U. (Animalcare group), who funded this project. JS, AM, JMC and GE declare no conflict of interest.

## AUTHOR CONTRIBUTION

JH and MS discussed and designed the PK studies described in these articles and wrote most of the manuscript. JS and AM designed and performed the protein binding and blood partitioning studies and developed the analytical methods used in the PK study. JMC and GE calculated and interpreted the pharmacokinetic parameters of both PK studies. All authors contributed to data interpretation and drafted parts of the manuscript. All authors approved the final manuscript.

## ANIMAL WELFARE AND ETHICS STATEMENT

The authors confirm that the animal housing and care of the studies described herein comply with the recommendations of Directive 2010/63/EU. Least numbers of animals were used in compliance with current regulations and scientific integrity. The welfare of the animals was taken into account in terms of number and extent of procedures to be performed. All study procedures had been checked and approved by the Animal Experimentation Ethics of the research centres involved.

## Supporting information

Supplementary Material

## Data Availability

The data that support the findings of this study are not shared due to confidentiality, but are available from the corresponding author upon reasonable request.
