# Supplementary material for: Pharmacokinetics of enflicoxib in dogs: Effects of prandial state and repeated administration
Source: J Vet Pharmacol Ther. 2021 Jun 23;44(6):888–901. doi: 10.1111/jvp.12995 (PMC9291756; doi:10.1111/jvp.12995)
Supplement: Supplementary file 1 — Supplementary Material [file JVP-44-888-s001.pdf]

Linear regression on C_{max} or AUC_{168} for week 1 an week 13 for enflicoxib and its pyrazol metabolite in healthy Beagle dogs treated with enflicoxib orally at a loading dose and once a week nominal doses of 8+4mg/kg (1X), 12+24 mg/kg (3X) and 40+20 mg/kg (5X).

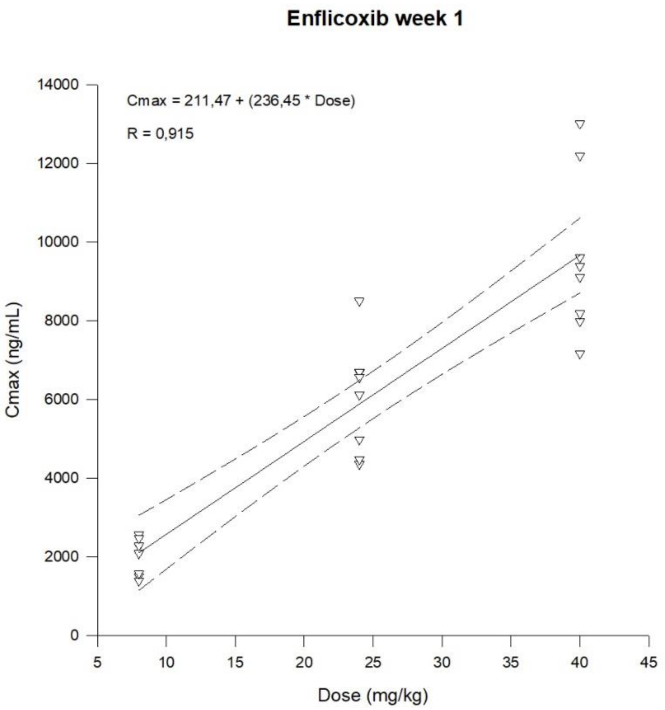

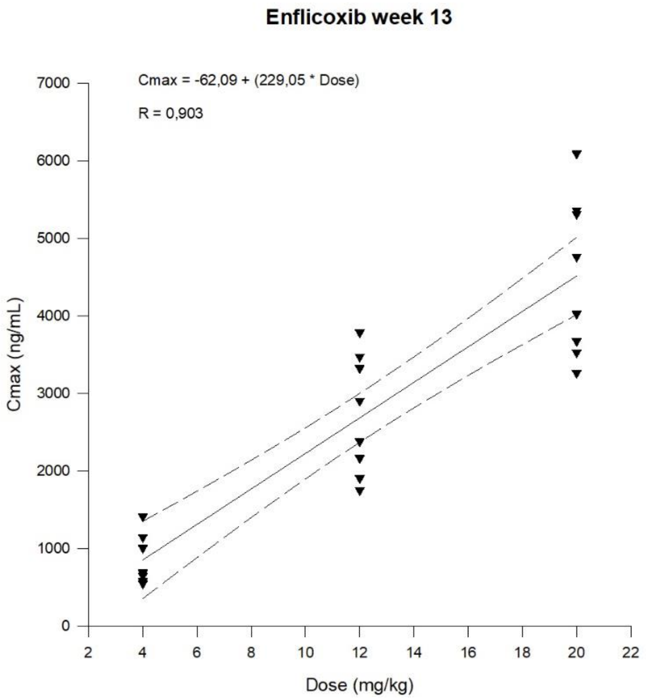

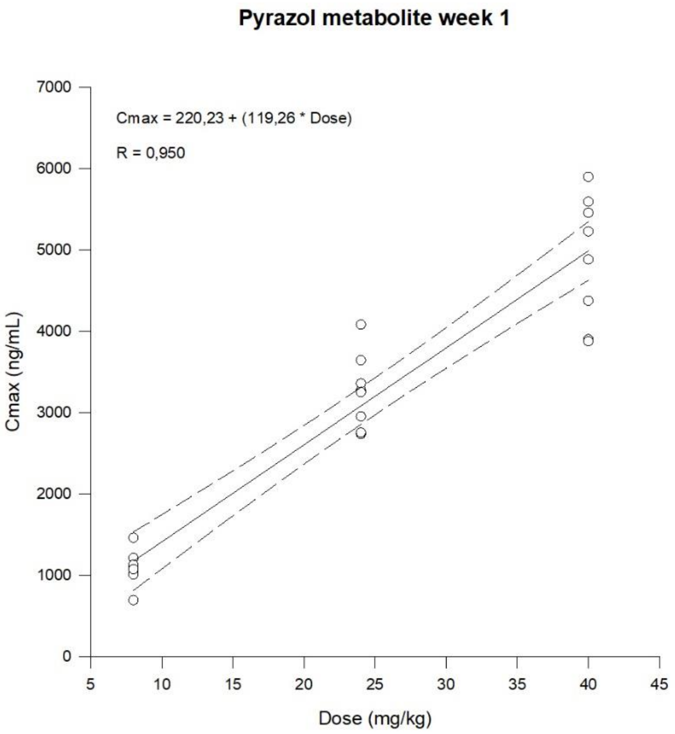

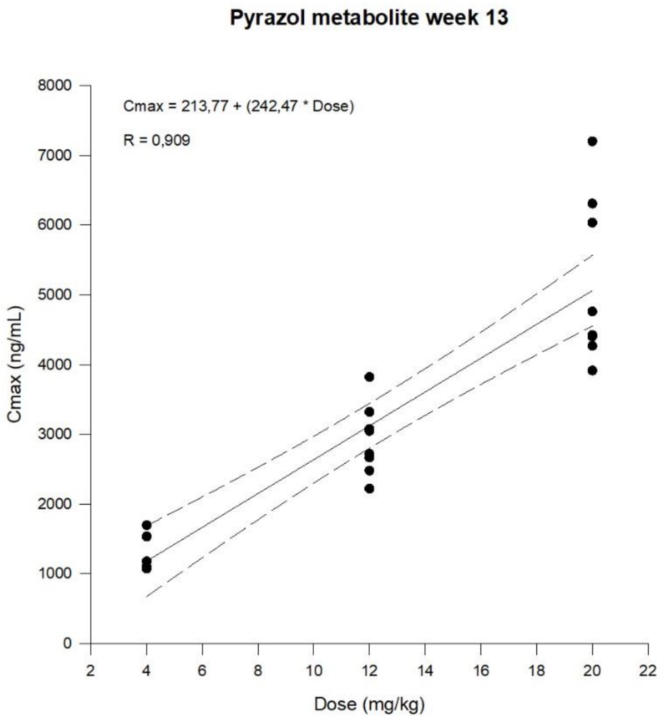

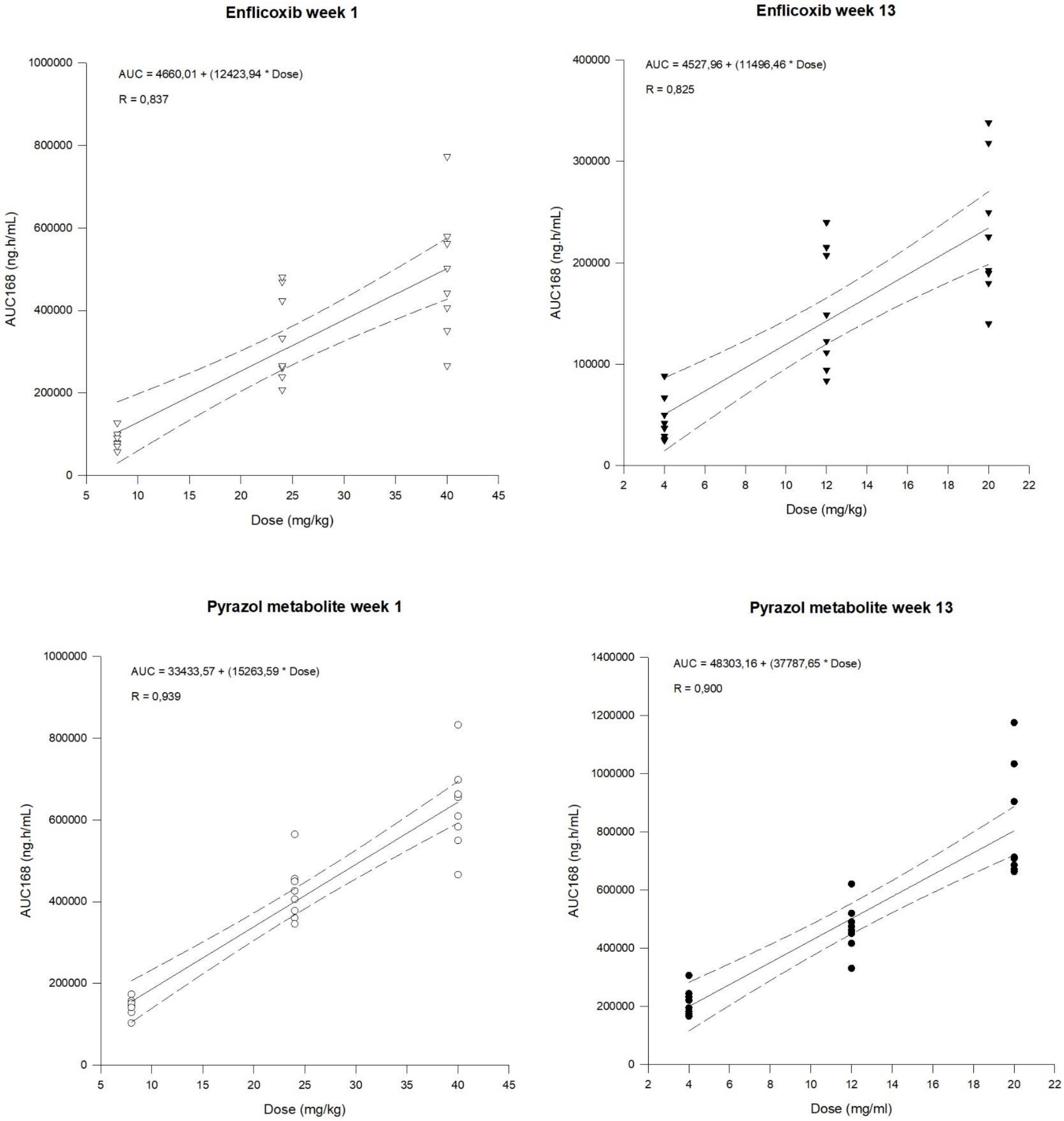
Enflicoxib week 1
AUC = 4660,01 + (12423,94 * Dose)
R = 0,837
AUC168 (ng.h/mL)
Dose (mg/kg)
Enflicoxib week 13
AUC = 4527,96 + (11496,46 * Dose)
R = 0,825
AUC168 (ng.h/mL)
Dose (mg/kg)
Pyrazol metabolite week 1
AUC = 33433,57 + (15263,59 * Dose)
R = 0,939
AUC168 (ng.h/mL)
Dose (mg/kg)
Pyrazol metabolite week 13
AUC = 48303,16 + (37787,65 * Dose)
R = 0,900
AUC168 (ng.h/mL)
Dose (mg/ml)
